# Juvenile Survival in a Neotropical Migratory Songbird Is Lower than Expected

**DOI:** 10.1371/journal.pone.0056059

**Published:** 2013-02-08

**Authors:** Matthew I. McKim-Louder, Jeffrey P. Hoover, Thomas J. Benson, Wendy M. Schelsky

**Affiliations:** 1 Department of Natural Resources and Environmental Sciences, University of Illinois at Urbana-Champaign, Urbana, Illinois, United States of America; 2 Illinois Natural History Survey, Prairie Research Institute, University of Illinois, Champaign, Illinois, United States of America; Universidad Nacional Autonoma de Mexico, Mexico

## Abstract

Attempts to estimate and identify factors influencing first-year survival in passerines, survival between fledging and the first reproductive attempt (i.e. juvenile survival), have largely been confounded by natal dispersal, particularly in long-distance migratory passerines. We studied Prothonotary Warblers (*Protonotaria citrea*) breeding in nest boxes to estimate first-year survival while accounting for biases related to dispersal that are common in mark-recapture studies. The natal dispersal distribution (median = 1420 m; *n* = 429) and a distance-dependent recruitment rate, which controls for effects of study site configuration, both indicated a pattern of short-distance natal dispersal. This pattern was consistent with results of a systematic survey for birds returning outside the nest box study sites (up to 30 km in all directions) within a majority (81%) of total available bottomland forest habitat, further suggesting that permanent emigration outside of the study system was rare. We used multistate mark-recapture modeling to estimate first-year survival and incorporated factors thought to influence survival while accounting for the potential confounding effects of dispersal on recapture probabilities for warblers that fledged during 2004–2009 (*n* = 6093). Overall, the average first-year survival for warblers reared without cowbird nestmates was 0.11 (95% CI = 0.09–0.13), decreased with fledging date (0.22 early to 0.03 late) and averaged 40% lower for warblers reared with a brood parasite nestmate. First-year survival was less than half of the rate thought to represent population replacement in migratory passerines (∼0.30). This very low rate suggests that surviving the first year of life for many Neotropical migratory species is even more difficult than previously thought, forcing us to rethink estimates used in population models.

## Introduction

Quantifying age-specific survival is necessary to identify factors affecting population growth and to model population dynamics. As juvenile survival is often thought to be lower and more variable than adult survival, estimating the mortality rate of juveniles can provide insights into reproductive tradeoffs and the evolution of life histories [1]–[3]. For birds, survival between fledging and reproduction (i.e. first-year survival in passerines) is an important life stage considered influential to population growth [4]–[6], yet it remains a “black box” of avian demography [2] because of the challenges associated with studying survival.

High mortality rates soon after fledging [7], [8] and natal dispersal typically confound efforts to accurately quantify first-year survival [9]. For many bird species, small body size prevents using radio-telemetry technology to estimate annual survival. Instead, mark-recapture methods are used to estimate survival of small avian species while accounting for imperfect detection [10]. However, one limitation of mark-recapture methods for estimating first-year survival is that there is no way to differentiate between permanent emigration and mortality [9]. As natal dispersal may lead to considerable rates of permanent emigration, particularly from study systems limited in size, first-year survival estimates are thought to be biased low.

The effects of natal dispersal on survival estimates may be particularly evident in migratory passerines, which annually fly vast distances between breeding and non-breeding locations. For example, typically <7% of migratory passerine nestlings banded in one year are resighted or recaptured within study populations in subsequent breeding seasons [11]. Based on the assumption that adult survival is approximately 0.60 for migratory passerines (reviewed in [12]), population modelers have generally used theoretical rates thought to represent population replacement, such as one-half of adult survival or ∼0.30 [13], [14]. Why then do studies commonly find local recruitment lower than the expected 0.30 value?

The natal dispersal distances (i.e. straight-line distance between fledging and first breeding locations) of migratory songbirds are thought to be greater than that of their non-migratory counterparts [15], [16], which may result in the increased probability of permanent emigration and reduced recruitment into their populations of origin. However, determining natal dispersal distances in migratory passerines has been difficult because of limitations in sample and study system sizes, and a general pattern of decreasing resight or recapture probabilities of dispersers with increasing distance, particularly when study areas are surrounded by vast available habitat [17]. Therefore, it remains unclear whether low juvenile return rates are caused by low survival or permanent emigration.

Incorporating the effects of dispersal into study design and statistical methodology is necessary to increase the accuracy of juvenile survival estimates [9], [18]. Likewise, accounting for dispersal is necessary to effectively investigate factors influencing first-year survival. One approach for dealing with the potential influence of dispersal on recapture probability is multistate modeling. This extension of the Cormack-Jolly-Seber model estimates the state-specific (e.g. location, reproductive status, behavior) probability of survival, recapture, and the likelihood of switching between states (transition probability) [19]. Because the detectability of an individual may vary as a function of numerous factors (e.g. time, age, gender, location), multistate modeling is a useful tool to account for potential biases generated by state-dependent recapture probabilities and uncertainty in state identity for occasions when the individual is not observed.

We estimated first-year survival of a Neotropical migratory passerine, the Prothonotary Warbler (*Protonotaria citrea*). First, using a long-term (1995–2010) breeding population we determined the distribution of natal dispersal distances. A distance-dependent recruitment rate was compiled to reduce confounds of nest box configuration on dispersal distances [20]. Further, to determine whether natal dispersal distances calculated from our study were a result of limitations associated with the size of the study system, we expanded the search for banded recruits by systematically searching outside the nest box study area (30 km in all directions) during 2008 and 2009. The habitat specificity of this species allowed us to systematically survey for dispersers within a majority of suitable breeding habitat. Then, by defining natal dispersal distances as states in a multistate framework, we studied several factors that may affect first-year survival while simultaneously controlling for the potential effects of natal dispersal distance on recapture probabilities. We examined whether recapture probabilities were influenced by natal dispersal distances, predicting that recapture probabilities would decrease with dispersal distance. Next, while accounting for potential effects of natal dispersal distances on resight or recapture probabilities, we included variables thought to influence survival rates: effects of season (fledging date), brood parasitism status (reared with or without a cowbird nestmate), nestling body condition, and brood size, to determine an overall first-year survival rate estimate for individuals in our study population.

## Methods

### Study area and species

**Figure 1:**
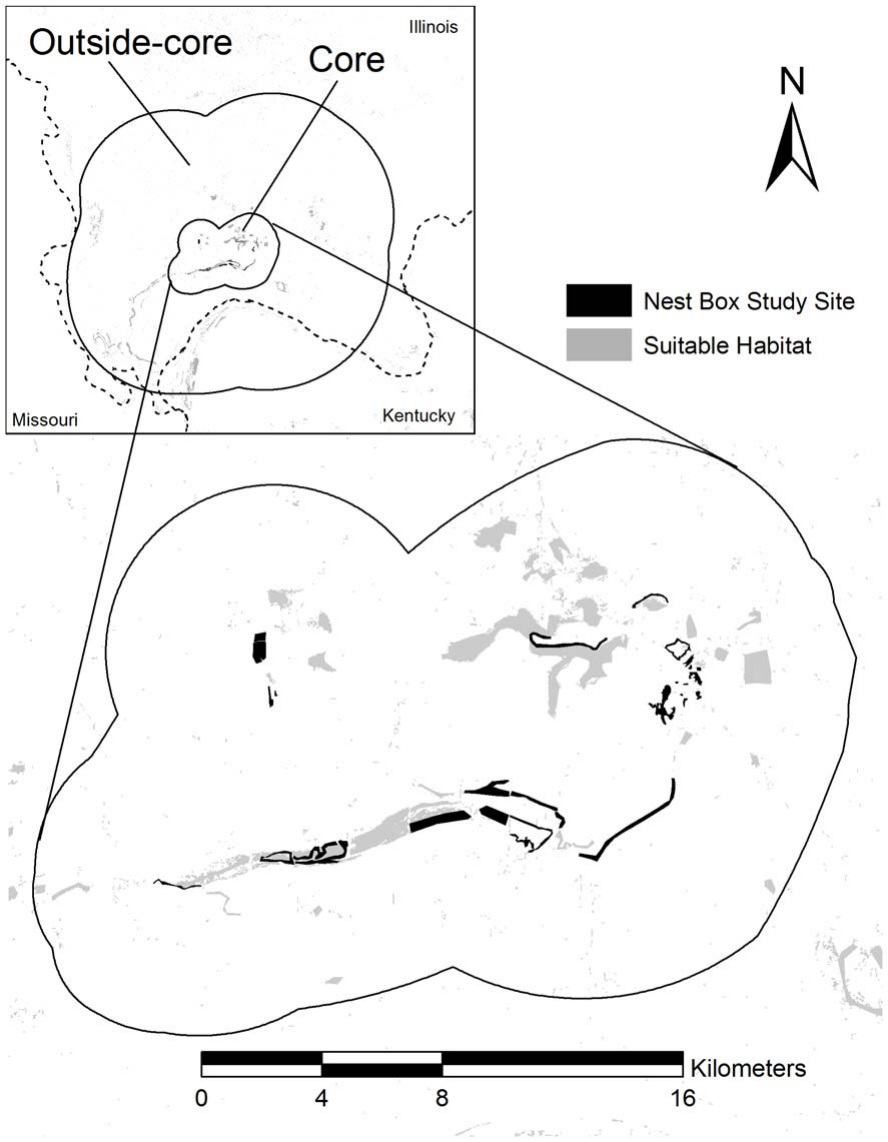
The entire study area depicting suitable habitat (light gray) determined by landcover data (Illinois State Geological Survey; Kentucky Geography Network) aerial photography and extensive surveys throughout the region. Black patches within core survey area indicate nest box study sites located in the Cache River watershed and dotted lines depict state boundaries.

The 4,875 km^2^ study area was located in southern Illinois and western Kentucky, U.S.A., and was divided into three regions: nest box sites, core, and outside-core areas (Figure 1) (see descriptions below). Prothonotary Warblers are long-distance migrants that winter in the Neotropics and breed in the eastern portion of the United States. These warblers are cavity nesters that breed almost exclusively near or over water within forested wetlands [21], and breed from late April to early August in our study area. Prothonotary Warblers readily use nest boxes when available and are commonly parasitized by an obligate brood parasite, the Brown-headed Cowbird (*Molothrus ater*) [22], [23].

### Data collection

During 1995–2010, we monitored approximately 1500 nest boxes distributed among 20–25 sites within an approximately 18 by 12 km area. Typically less than half of the nest boxes were used in a given year, suggesting that nest sites were not limiting. Within each site, we placed nest boxes 40–50 m apart within appropriate habitat. Nest boxes were attached to trees, placed 1.7 m above ground and had 44-mm-diameter openings, similar to the attributes of natural cavities used by warblers in this study system [24]. From 1999–2010, a majority of the nest boxes were removed from trees and attached to greased conduit poles to reduce nest predation. We monitored nest boxes every 3–6 days throughout the breeding season and recorded the number of warbler and cowbird eggs and nestlings present each visit. Prior to fledging (age 5–8 days), we banded each nestling's right leg with a uniquely numbered aluminum U.S. Geological Survey band, and measured mass (±0.25 g) and tarsus length (±0.5 mm). We assumed nestlings fledged if they reached 10–11 days of age and the nest was empty and intact. Additional evidence of fledging included the presence of trampled droppings in the nest, alarm calls from adults, and observations of appropriately aged fledglings in the territory. Nestlings that did not survive to fledge were not used in analyses.

We identified banded recruits, individuals banded as nestlings that returned to breed in a subsequent year, as those individuals having a single aluminum band on the right leg. Once captured, we determined the origin of each banded recruit and determined their dispersal distance by measuring the straight-line distance between natal nest box and first recapture location. Nest boxes and recapture locations were recorded with a global positioning system (GPS) unit or identified on topographic maps, accurate to approximately 25 m. Male recruits were captured using audio playback with a decoy placed next to a mist-net and female recruits were captured by placing a small plastic bag over the nest box opening while they were incubating. We marked all banded recruits and other breeding adults with unique combinations of colored leg bands. We assigned adults to active nests based on territorial behavior and their presence at individual nest boxes; each year we knew the identity of >95% of the adults on each nest box study site.

#### Systematic survey for banded recruits off nest box study sites

In addition to the information collected from the nest box study sites, we conducted a systematic survey between 15 May and 4 July in 2008 and 2009 to locate banded recruits of all ages outside of the nest box study sites. We defined the core survey area as all suitable breeding habitat located in the areas between nest box sites and within a 5-km buffer surrounding the nest box sites (Figure 1). Suitable breeding habitat located from 5 km to 30 km surrounding the core survey area was defined as outside-core (Figure 1). We used our knowledge of the region, topographic maps, aerial photography, and landcover data from Illinois State Geological Survey (www.isgs.uiuc.edu) and Kentucky Geography Network (kygeonet.ky.gov) to locate suitable breeding habitat within each survey area. We used ArcMap 9.1 (ESRI 2005) to estimate the proportion of total suitable habitat (km^2^) surveyed.

In each of the two years, we broadcasted male songs to survey for banded recruits within appropriate breeding habitat. At approximately 75-m intervals throughout appropriate habitat, songs were played for one minute or until an individual approached and was identified. We used binoculars to observe the legs of responsive adults to determine if they were banded. We noted the location of other nearby Prothonotary Warblers (e.g. singing males and chipping females) to reduce the chance of re-counting unbanded adults. Because females are less responsive to playback, we attempted to locate and determine the banding status of females first when pairs responded to playback. Individuals with a single aluminum band were designated as banded recruits and were subsequently captured. We placed a single yellow color-band on the left leg of banded recruits captured outside of the nest box study sites to eliminate the chance of double-counting individuals within the same survey. Banded recruits captured in the previous year, as identified by the single-yellow-plastic and aluminum band combination, were noted during the 2009 systematic surveys. In each of the two years of systematic surveys, we calculated the proportion of banded recruits within the surveyed breeding population for both the core and outside-core areas. Any one-year-old banded recruits recaptured during the systematic surveys were included in analyses of natal dispersal distances.

#### Detection probabilities of systematic survey

Our systematic survey could be biased if the probability of locating banded recruits varies with increased distance from the nest box area. To test for this bias, we used the systematic survey playback protocol to conduct repeated surveys at six sites (core: *n* = 3; outside-core: *n* = 3) in 2009. The survey sites were similar in size (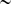
30 ha) and number of adults detected (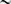
15), and were all separated by >1 km. We returned to each site on three occasions separated by at least one week. Using program MARK [25], we used occupancy modeling [26] to determine if detection probability varied between survey areas.

### Distance-dependent recruitment rate

The distances between study sites and number of birds produced (and banded) per site can bias the natal dispersal distribution [27]. A distance-dependent recruitment rate (DDRR) compares the number of recruits relative to the number banded within that distance class, thereby limiting the effects of the configuration and productivity of the study sites on the resulting distribution of natal dispersal distances [20]. To calculate the DDRR we used the methods outlined in [20]. For each banded recruit, we determined the numbers of nestlings banded during the fledging year of the recruit for several distance categories relative to the fledging location of the recruit (in 2 km classes). When combined for all recruits, we calculated the average number of nestlings banded for each distance class. The observed number of recruits was then divided by the number of nestlings banded in the relative distance class to create the annual DDRR, or number of recruits observed for each nestling banded in the relevant distance class. Annual DDRRs were averaged and weighted by the number of recruits per year and are presented with standard errors. Because we were interested in corroborating the distribution of natal dispersal distances within the nest box study sites, only one-year-old banded recruits that returned to a nest box were used in this analysis.

### Multistate mark-recapture analysis

We estimated first-year survival for warblers that fledged during 2004–2009 in the Cache River watershed nest box study sites. Nestlings that fledged prior to 2004 lacked measurements of nestling condition (see model covariates) and were not included in the survival analysis. To account for the possibility that recapture probability declines with increasing natal dispersal distance, we used multistate mark-recapture models [19] to incorporate the transition of individuals from fledging to one of four distance categories (<2 km, 2–4 km, 4–6 km, and >6 km) in a subsequent breeding season. Like typical Cormack-Jolly-Seber models, multistate mark-recapture allows for the estimation of survival probability (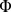) that accounts for imperfect resight/recapture probability (*p*). However, these models provide the added flexibility of incorporating discrete states, accounting for transitions among states (Ψ), uncertainty in state membership for occasions when an individual was not observed, and estimates of survival probability and resight/recapture probability that are specific to each state. In our case, we used distance categories as discrete states. In each individual encounter history, we classified observations as one of six states: state 1 was the initial marking prior to fledgling; states 2–5 included local recruitment into one of four dispersal distance categories; and state 6 was an ‘absorbing state’ representing individuals resighted or recaptured in breeding seasons after their initial recapture occasion. For example, an encounter history of 0126600 indicates the nestling was initially marked in 2005, recaptured <2 km from the nest in which it was banded (i.e. state 2) in 2006, and relocated again in 2007 and 2008. Individuals were constrained to transition from state 1 (fledgling) to one of the four distances categories (states 2–5), and to state 6 thereafter. There were no transitions among distance categories (states 2–5), to state 1, or out of state 6. We focused on resight/recapture, transition, and survival probabilities during the first year by using two age classes, first-year and adult, using a time-since-marking approach.

#### Model selection and goodness-of-fit

To minimize the number of models we considered, we used a three-step approach. First, we evaluated models that varied transition probability while maintaining age-dependent survival probability (i.e., first-year vs. adult), and age- and state-dependent resight/recapture probabilities. Using the top-ranked transition probability structure and age-dependent survival probability, we considered models that varied resight/recapture probabilities. In the final step, the best transition and resight/recapture probability structures were used while evaluating models that varied in survival probability. We assessed the goodness-of-fit (GOF) of our models using program U-CARE [28]. We performed multistate mark-recapture analyses in program MARK [25] and used SAS (SAS version 9.2; SAS Institute 2008) for all other analyses. Model selection was based on Akaike's Information Criterion adjusted for small sample sizes and overdispersion (QAIC_C_) and we used model averaging to account for model-selection uncertainty and to present parameter estimates [29].

#### Model covariates

We evaluated the influence of four variables on survival probabilities: the number of warbler nestlings reared within the brood (range 1–6), presence of a cowbird nestmate (yes or no), fledging date (ordinal date), body condition, and an interaction between parasitism status and fledging date. We used residuals from a regression of body mass and tarsus length as an index of nestling body condition [30]. We projected the fledging date of each individual by estimating the nestling age during banding and assumed fledging at 10 days old [21]. We were unable to determine the sex of nestlings at the time of banding and thus excluded sex from the survival analysis. Variables were not highly correlated (|*r*|<0.70). Explanatory variables were considered important if their 95% confidence interval excluded zero. To ensure covariate effects were not generated by variation in detectability, we explored models incorporating the same variables as covariates for resight/recapture probability. We present all parameter estimates with 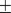
1 standard error (SE) and survival estimates are derived from model averaging.

This study was carried out in strict accordance with the recommendations in the Guidelines to the Use of Wild Birds in Research (Available: http://www.nmnh.si.edu/BIRDNET). Research was approved by the University of Illinois Institutional Animal Care and Use Committee (Permit Numbers: 04092 and 10173), the U.S. Fish and Wildlife Service (Permit Number: MB815400-0), and the U.S. Geological Survey (Banding Permit Number: 06507).

## Results

### Natal dispersal distance distribution

**Figure 2:**
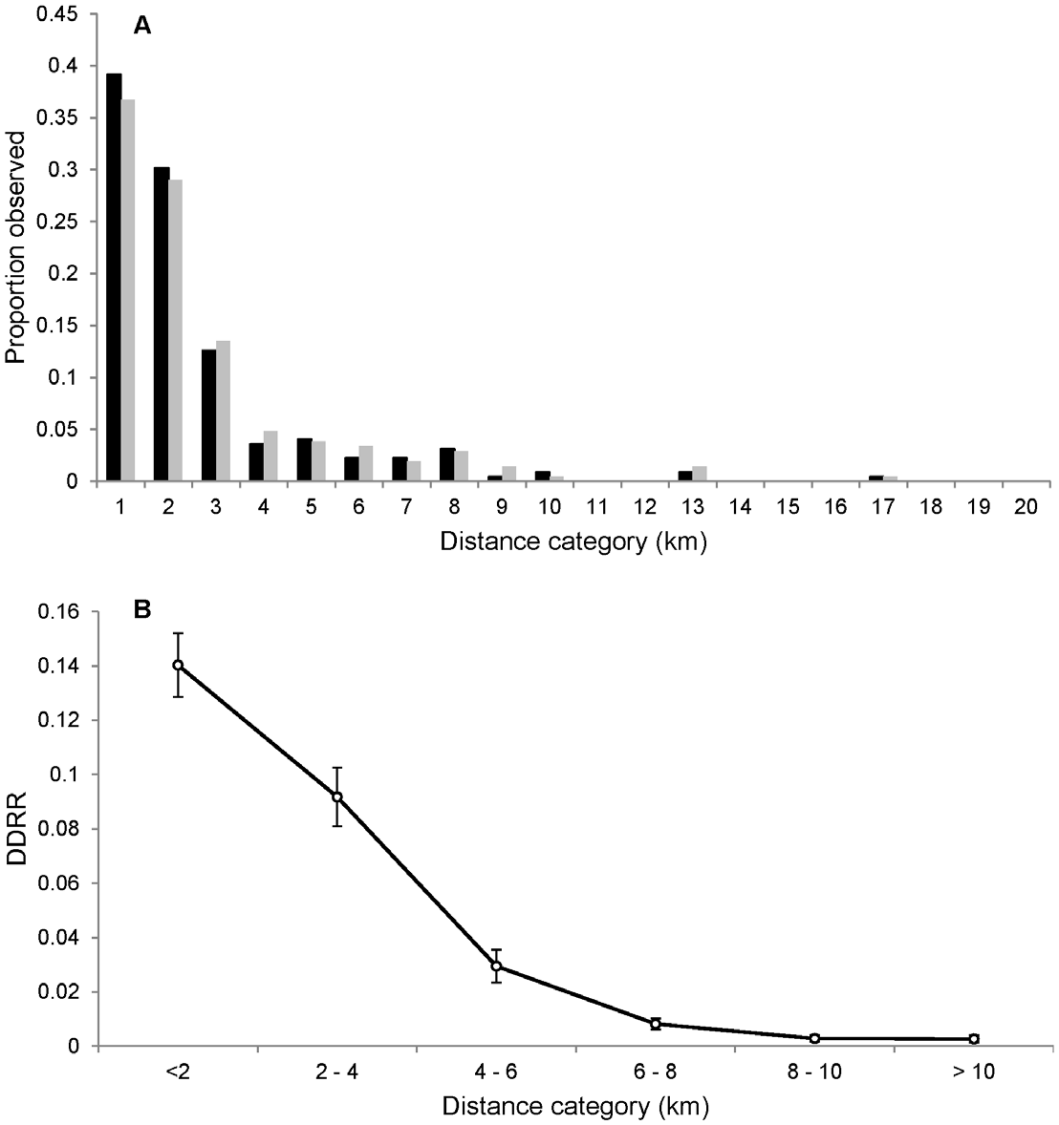
The distribution of natal dispersal distances for one-year-old Prothonotary Warblers in southern Illinois, fledging during 1995–2009 and recaptured on nest box study sites and within the systematic survey area. (A) The observed natal dispersal distance distribution for 222 female (black) and 207 male (gray) and (B) the distribution of observed natal dispersal distances relative to the number banded in that distance class (DDRR). Mean weighted DDRR and SEs for individuals banded prior to fledging are presented.

Of the 9,289 nestlings banded prior to fledging during 1995–2009, 429 one-year-old banded recruits were captured and 250 banded recruits were first captured when they were two years old or older (total = 679, 7.3%). The median natal dispersal distance of one-year-old banded recruits (*n* = 429) did not differ between the sexes (*U*-test, *z* = 0.78, d.f. = 1, *P* = 0.43; Figure 2A), therefore we pooled across sex to derive the distribution of natal dispersal distances. The overall median dispersal distance was 1.42 km and the distribution of all natal dispersal distances was skewed and leptokurtic (skewness = 2.68; kurtosis = 9.00). Similarly, the mean DDRR was greatest within the <2 km distance class (0.14), and decreased with increasing distances (Figure 2B) reflecting a pattern of short-distance natal dispersal (see Figure 1d in [20]). If the pattern of natal dispersal was in fact random or long-distance in this population, the DDRR would have been either a flat or negatively-skewed curve, respectively, across distance classes.

### Systematic survey for banded recruits off nest box study sites

Approximately 89% of all resighted banded recruits located off the nest box study sites during 2008 and 2009 (*n* = 75) were recaptured and identified. Although a greater amount of suitable habitat occupies the outside-core (25.5 km^2^) versus core survey areas (9.65 km^2^), we surveyed approximately 81% of suitable habitat and there was no significant difference in the proportion of suitable habit surveyed between each survey area (χ^2^ = 0.33, d.f. = 1, *P* = 0.56). More adults were examined in the outside core (2008, *n* = 717; 2009, n = 968) than within the core survey area (2008, *n* = 477; 2009, *n* = 473). The proportion of surveyed adult warblers that were banded recruits was significantly greater within the core survey area (10%) than the outside-core area (0.1%) (χ^2^ = 156.80, d.f. = 1, *P*<0.001). We failed to detect a significant year effect on the proportion of observed banded recruits in either survey area (core; χ^2^ = 1.43, d.f. = 1, *P* = 0.23, outside-core; χ^2^ = 2.70, d.f. = 1, *P* = 0.10). Only two banded recruits were detected in the outside-core survey area; both individuals were observed separately 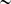
5.25 km from the nearest nest box study site. Within the core survey area, more males (2008, *n* = 400; 2009, *n* = 407) were observed than females (2008, *n* = 77; 2009, *n* = 66), yet the proportion of surveyed birds that were banded recruits did not differ between the sexes (males = 10.7%, females = 10.4%, χ^2^ = 0.10, d.f. = 1, *P* = 0.75).

#### Detection probabilities of systematic survey

Our best supported occupancy model indicated that the detection probability of banded recruits averaged 0.89 (95% C.I. = 0.49–0.98) and did not differ between the core and outside-core survey areas. Furthermore, the relatively high estimate of detection probability of banded recruits (0.89) supports a single visit to each patch of suitable habitat was sufficient to locate most banded recruits and to allow for comparisons between core and non-core areas.

### First-year survival

We analyzed the encounter histories of 6,093 individuals banded as nestlings (2004–2009), of which, 418 individuals were recaptured in a subsequent year. Although we were unable to determine sex prior to fledging, similar numbers of males (*n* = 212) and females (*n* = 206) were recaptured. The test for GOF indicated some lack of fit between the data and the JollyMove (JMV) model (χ^2^ = 23.44, d.f. = 14, *P* = 0.05). This lack of fit was caused by lower numbers of resights or recaptures in the following year (χ^2^ = 16.05, d.f. = 3, *P* = 0.001) which was confirmed by running the GOF test while suppressing the first encounter for each individual (χ^2^ = 7.03, d.f. = 12, *P* = 0.86). Consequently, we proceeded with fitting models with a time-since-marking structure that incorporated different survival and resight/recapture probabilities between the first and subsequent recapture periods. To reduce potential effects of overdispersion, we incorporated an estimated variance inflation factor (*ĉ* = 1.67) based on the sum of the GOF tests in U-CARE (calculated as χ^2^ divided by the degrees of freedom).

#### Transition probabilities

Models with a two-stage structure (State 1→States 2–5, and States 2–5→State 6) had much greater support than the constant model (Δ QAIC_C_ = 139.62). There was little support for annual variation in transition probabilities (Δ QAIC_C_ = 5.87). The transition model with the greatest support (*w_i_* = 0.99) incorporated variation from state 1 to each distance-specific state (2–5), while the transitions between states 2–5 to the ‘absorbing’ state 6 were held constant (Table S1). Transition probabilities decreased dramatically with distance, with the highest probability of local recruitment within the <2 km distance category (0.68
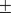
0.02; Figure S1). This transition structure was used in subsequent modeling of recapture probabilities and survival rates (Table S1).

#### Recapture probabilities

Contrary to expectations, recapture probabilities from this study did not decrease with increasing dispersal distance. In a model that included variation in first-year recapture probabilities among distance categories (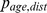), recapture probabilities declined slightly from the first distance category (<2 km; 0.45
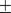
0.03) to the second (2–4 km; 0.36
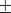
0.07), but increased within state 3 (4–6 km; 0.53
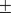
0.14). Because distance-related first-year recapture probabilities were not supported (*w_i_*<0.01), we only used models incorporating annual variation for subsequent analyses (Table S2). The top-ranked recapture model incorporated an effect of year on first-year recapture probabilities and constant probability for ages >1 year old (*w_i_* = 0.98; Table S2). Recapture probability for first-year warblers varied between 0.56 (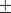
0.06) and 0.28 (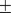
0.05) among years.

#### Survival probabilities

Incorporating the top-ranked transition and recapture structures, models that included two age-classes in survival probability estimates were better supported than a constant model (ΔQAIC_C_ = 39.88). Annual variation in first-year survival probability was not supported when compared to the age-class model (Table 1). Thus, individual covariates were applied to a two age-class model that included time-constant survival probability estimates.

**Table 1:** Model selection to estimate first-year apparent survival for Prothonotary Warblers, *Protonotaria citrea*, in southern Illinois, USA, fledging during 2004–2009.

No.	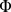	QAIC_C_	ΔQAIC_C_	*w_i_*	*K*
Models without effect of cowbird parasitism on first-year apparent survival					
1	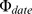	4265.01	5.48	0.01	15
2	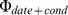	4265.44	5.91	0.01	16
3	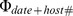	4266.34	6.81	0.00	16
4	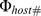	4296.37	36.84	0.00	15
5	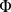	4299.40	39.88	0.00	14
6	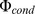	4299.62	40.01	0.00	15
7	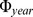	4303.56	44.04	0.00	19
Modeling the effect of cowbird parasitism on first-year apparent survival					
8	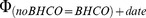	4259.53	0.00	0.23	16
9	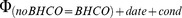	4259.99	0.47	0.18	17
10	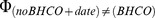	4260.13	0.60	0.17	16
11	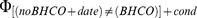	4260.50	0.97	0.14	17
12	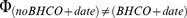	4261.30	1.78	0.09	17
13	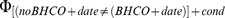	4261.74	2.22	0.07	18
14	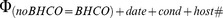	4261.92	2.39	0.07	18
15	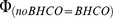	4297.73	38.21	0.00	15
16	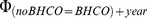	4306.83	47.30	0.00	24

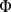
, apparent survival; QAIC_C_, quasi-likelihood Akaike's information criterion corrected for small sample size; *w_i_*, Akaike's model weights; *K*, number of parameters; *date*, ordinal fledging date; *BHCO*, reared with cowbird nestmate; *noBHCO*, absence of cowbird nestmate; *cond*, nestling body condition; *host*#, number of warbler nestmates within brood;  = , indicates no interaction between terms; ≠, indicates an interaction between terms; year, annual variation; (.), indicates a constant for parameter.

**Figure 3:**
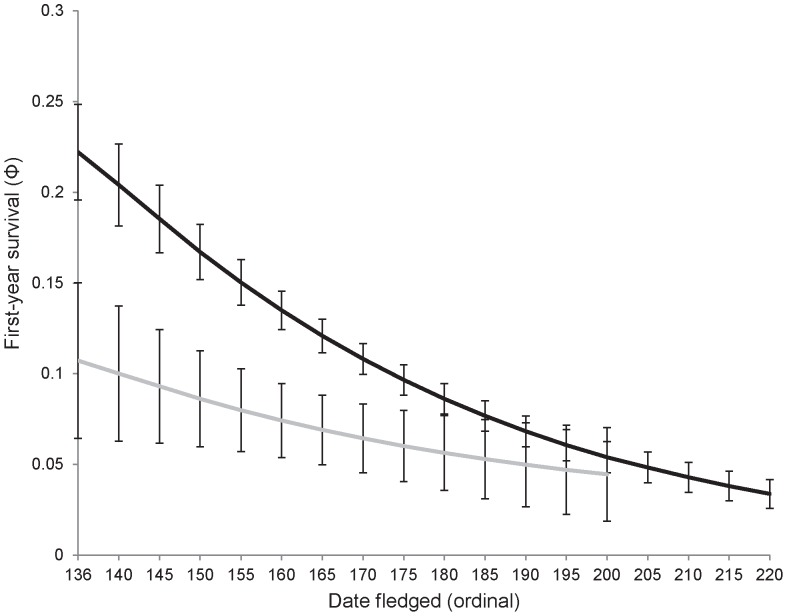
The relationship between fledge date (ordinal fledging date 136 = 15 May) and first-year survival for Prothonotary Warblers. Model averaged estimates (mean 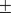 1SE) of warblers reared with Brown-headed Cowbirds (grey line) and in the absence of cowbirds (black line) in southern Illinois, USA, fledging during 2004–2009 are presented. All other variables were held at mean observed values.

First-year survival estimates varied as a function of fledging date and parasitism status (Table 1). The top ranked model (Model 8; Table 1), included similar linear trends for the effect of fledging date on individuals reared with a cowbird nestmate (BHCO) and without (noBHCO). Overall, all models <10 ΔQAIC_C_ included fledging date, and model averaged estimates of first-year survival declined with increasing fledging dates both for individuals reared with and without a cowbird nestmate (Figure 3). Similarly, while holding other covariates at mean observed values, model averaged survival estimates were nearly 2 times greater for individuals reared without cowbirds (0.11
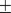
0.01) than reared with a cowbird nestmate (0.06
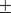
0.01) (Figure 3). An interaction between the effects of fledging date and cowbird parasitism on first-year survival were marginally supported. The model fit was slightly improved by removing the effect of date for group BHCO (Table 1; Models 10 and 11) and the predicted model-averaged estimates indicated that first-year survival decreased with fledging date less sharply for individuals reared with a cowbird nestmate (BHCO) than those without (noBHCO) (Figure 3).

Variation in nestling body condition (*cond*) was only marginally supported to influence first-year survival. Despite a model incorporating condition having nearly equal support to the top-ranked model (Model 9; ΔQAIC_C_ = 0.47), the 95% CI overlapped zero (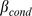 = 0.09, 95% CI: −0.05 to 0.23) and the model including this covariate alone (Model 6) was not supported. The effect of number of warblers within the brood (*host#*) on first-year survival was unimportant (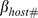 = 0.01, 95% CI: −0.14 to 0.16). When the survival covariate structures were interchanged with the recapture probabilities, QAIC_C_ decreased by >2, suggesting that variation in survival estimates was not being driven by the effects of explanatory variables on recapture probabilities.

## Discussion

In lieu of reliable estimates of first-year survival, population modelers have used theoretical values thought to represent adequate population-level replacement rates, such as one-half of adult survival, or ∼0.30 ([13], [14] reviewed by [12]). In contrast, we found both the mean (0.11
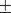
0.01) and maximum (early fledged; 0.22
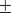
0.03) first-year survival estimates for non-parasitized Prothonotary Warblers to be much less than the expected rate of first-year survival for a migratory passerine.

The Cache River nest box study system provided a rare opportunity to investigate natal dispersal and ultimately first-year survival for a migratory passerine, as the nest boxes allowed for a large sample size and the habitat specificity of the warblers enabled us to focus our search for banded recruits. While exciting new statistical methods are being developed to account for the rate of permanent emigration [9], [31], the multistate mark-recapture modeling allowed for variable resight/recapture probabilities as a function of distance and minimized the traditional biases inherent to these types of studies. Our systematic surveys for natal dispersal events outside of the nest box study system, distribution of natal dispersal distances, and the distance-dependent recruitment rate all suggested that permanent emigration was relatively rare in our study system. Survival would be underestimated if long-distance natal dispersal (i.e. outside of systematic survey area) were common in this population. However, Winkler et al. [32] summarized data from one of the largest study areas and sample sizes for a Neotropical migrant to date and also found that long-distance natal dispersal occurs rarely (1.3% of observed dispersal events at 50–200 km) while the majority of first-year Tree Swallows (*Tachycineta bicolor*) returned to breed within 10 km (median = 2.8 km) of their natal origin. Although Prothonotary Warblers dispersing (i.e. permanently emigrating) off the nest box sites were detected in our systematic surveys, survival estimates and resight/recapture probabilities during the years of systematic surveys did not increase. Even though apparent survival estimates always represent a minimum value for the true estimate, we believe that by accounting for dispersal and using multistate mark-recapture models that incorporate factors influencing survival and resight/recapture probability, including distance, we calculated robust estimates of first-year survival.

For populations with low juvenile survival, relatively high adult survival or fecundity would be required to maintain population stability. As adults tend to disperse between years after experiencing nesting failure, adult survival estimates in migratory passerines (i.e. 0.60) would likely be increased with the incorporation of reproductive performance [18]. For example, experimental manipulations of reproductive success randomly assigned to Prothonotary Warblers led to the discovery that adult return rates in double-brooded individuals is approximately 0.80 [33]. As a return rate is a minimum estimate of survival, adult survival for Prothonotary Warblers is likely greater than 0.80 and low juvenile survival (i.e. 0.11) may be offset by very high adult survival. Using these survival rates (0.11 for juveniles and 0.80 for adults) and a simple population model [λ = adult survival+(fecundity×juvenile survival)], a fecundity value of 1.82 would be necessary to achieve population stability (i.e. λ = 1.00). Indeed, during our many years of working in this study system, fecundity values have often met or exceeded the value necessary for populations to maintain themselves in the watershed [24], [34], [35]. In addition, the relatively short median natal dispersal distances (e.g. <2 km) we observed strongly suggests that local conservation and habitat management efforts to increase nesting success of this species will have positive effects on local breeding populations.

Juvenile survival estimates in migratory species are exceedingly rare, but a few studies have projected first-year survival estimates by combining post-fledging survival rates with survival estimates documented within the breeding and non-breeding areas [36] and also reported values well below 0.30: between 0.18 and 0.24 (Lark Bunting, *Calamospiza melanocorys*) [8]; and between 0.15 and 0.18 (Black-throated Blue Warbler, *Setophaga caerulescens* [37]). While relatively low juvenile survival may be representative of many migratory songbird populations, other recent estimates have indicated that juvenile survival is variable and may reflect differences in life history traits [1], [3], [38], [39]. For example, juvenile survival estimates in two aerial insectivores were more than twice as high as the estimate found for Prothonotary Warblers (Purple Martin, *Progne subis* = 0.27 [40]; Eastern Kingbird, *Tyrannus tyrannus* = 0.29 [41]) and may reflect differences in how these species experience the first weeks of the post-fledging period. Purple Martins are fully capable of extended flight when they fledge and spend much of the time foraging while in flight [40], and Eastern Kingbirds can sustain short flights during the early part of the post-fledging period [42]; in each case resulting in very low post-fledging mortality rates. In contrast, recently-fledged Prothonotary Warblers are poor flyers, not very mobile, and still highly dependent on their parents, possibly making them more vulnerable to predators during this period. Our estimate of low juvenile survival (i.e. 0.11) in Prothonotary Warblers may not be generalizable to all migratory passerines, but may represent what juvenile survival is in other forest-dwelling Neotropical migrants. Additional studies that combine intensive efforts to locate returned juveniles over a large area with new and emerging modeling and analysis techniques to generate estimates of juvenile survival in other species will clarify whether our value of 0.11 is more an exception or a general rule.

Considerable variation in first-year survival rates were observed with the inclusion of biotic factors into our survival models. The probability of first-year apparent survival was on average 40% lower for those reared with a parasitic cowbird nestmate than for those reared with only host nestmates. Despite fledging from the nest, the inability of host young to adequately compete with brood parasites for food during the nestling stage [43], [44] may increase the probability of mortality post fledging. However, our measurements of body condition for nestlings did not appear to explain the observed decrease in survival, regardless of parasitism status. There may be other negative effects of cowbirds not measured in this study (e.g. reduced immune function [45]) that reduce survival rates for individuals reared with cowbird nestmates. In addition, brood parasites likely continue to disproportionally procure resources during the post-fledging period, potentially reducing body condition further and thereby reducing survival prior to independence for hosts [46], [47]. Competition for food between host and parasitic fledglings could leave host fledglings in a weakened condition and less able to escape from predators or cause them to increase their begging only to attract more predators [48].

The probability of first-year survival decreasing with later fledging dates (i.e. a seasonal effect) has been reported in populations of resident species (reviewed in [49]), and recently in migratory passerines [40], [50]. First-year survival estimates for non-parasitized warblers decreased from 0.22 (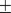
0.02) to 0.03 (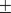
0.03) for fledge dates across the breeding season, with a substantial reduction during the first month (0.12). Parental quality and seasonal variation in ecological factors (e.g. food limitation, parasites, predation), two common hypotheses explaining temporal variation in reproductive performance [49], may also explain why first-year survival rates decreased with later fledging dates. In migratory birds, adults of ‘high quality’ are thought to arrive on the breeding grounds earlier and subsequently initiate breeding prior to individuals of ‘lower’ quality [51]. However, the parental quality hypothesis alone fails to explain the dramatic decline in first-year survival with increasing fledging dates found in this study. A majority (>65%) of the adult females fledging offspring late in the season were known to have also bred earlier (April and May) within the same year. If it were simply parental quality driving the seasonal decline, first-year survival probabilities in late-fledged birds would likely be much greater because most ‘high quality’ individuals (i.e. early breeding birds) bred a second time. As a substantial portion of first-year mortality likely occurs during the post-fledging stage (reviewed in [12]), the influence of food availability [8] or intensity of predation [48] may increase as the breeding season progresses, thus reducing survival of fledglings prior to migration. Finally, lacking the ability to use previous migratory movements for navigation, juveniles may incur a greater risk of mortality during fall migration than adults [39]. Individuals that fledge earlier in the breeding season may benefit from having additional time to adequately prepare for migration (e.g. fat reserves), thereby increasing the probability of successfully reaching the wintering grounds [50].

The very low juvenile survival found in this population suggests that mortality rates during the first year of life for many Neotropical migrants are potentially greater than previously thought. Brood parasitism and timing of reproduction are important effects on first-year survival and, subsequently, provide insights into potential areas of vulnerability in populations of conservation concern. Estimates used in past population models (e.g. one-half of adult survival) are unlikely to reflect first-year survival for all migratory passerine populations, and future population models should incorporate a range of first-year and adult survival rates. Furthermore, current estimates of adult survival in migratory songbirds are likely biased low and future research should incorporate reproductive success into survival models to account for permanent emigration after reproductive failure [17]. In study systems where banded recruits cannot be searched for systematically, juvenile survival could be estimated by incorporating the dispersal distribution within a mathematical framework to determine the rate of permanent emigration [8], [30]. Increasing the accuracy of age-specific survival estimates is necessary to enhance our understanding of population dynamics, tradeoffs in reproduction and the evolution of avian life histories.

## Supporting Information

Figure S1Probability (mean 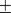 1 SE) of transition between fledging and four distance categories for Prothonotary Warblers in southern Illinois, USA, 2004–2010.(TIFF)

Table S1 Model selection to estimate transition probabilities for Prothonotary Warblers, *Protonotaria citrea*, in southern Illinois, USA, 2004–10.
(DOCX)

Table S2 Model selection to estimate recapture probabilities for Prothonotary Warblers, *Protonotaria citrea*, in southern Illinois, USA, 2004–10.
(DOCX)
