# Supplementary figures and images for: Juvenile Survival in a Neotropical Migratory Songbird Is Lower than Expected
Source: PLoS One. 2013 Feb 8;8(2):e56059. doi: 10.1371/journal.pone.0056059 (PMC3568049; doi:10.1371/journal.pone.0056059)

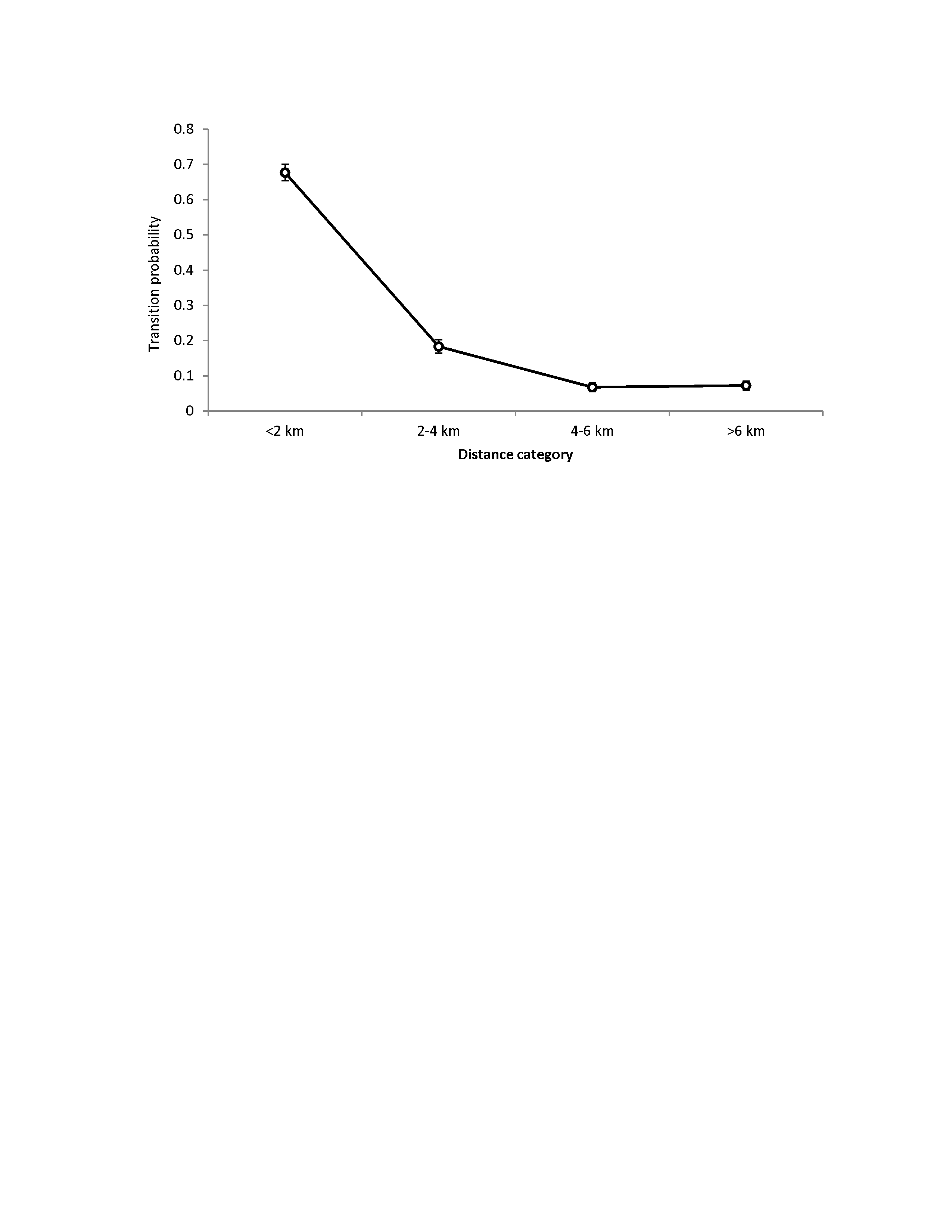

Supplement: Figure S1 — Probability (mean 1 SE) of transition between fledging and four distance categories for Prothonotary Warblers in southern Illinois, USA, 2004–2010. (TIFF) [file pone.0056059.s001.tiff]
