# Supplementary material for: Juvenile Survival in a Neotropical Migratory Songbird Is Lower than Expected
Source: PLoS One. 2013 Feb 8;8(2):e56059. doi: 10.1371/journal.pone.0056059 (PMC3568049; doi:10.1371/journal.pone.0056059)
Supplement: Table S1 — Model selection to estimate transition probabilities for Prothonotary Warblers, Protonotaria citrea, in southern Illinois, USA, 2004–10. (DOCX) [file pone.0056059.s002.docx]

Table S1. Model selection to estimate transition probabilities for Prothonotary Warblers, *Protonotaria citrea*, in southern Illinois, USA, 2004-10.

| $\Phi$ | $p$ | Ψ | QAIC_C_ | ΔQAIC_C_ | *w_i_* | *K* |
| --- | --- | --- | --- | --- | --- | --- |
| $\Phi_{age}$ | $p_{age,dist}$ | $\Psi_{age,dist}$ | 4309.01 | 0.00 | 0.99 | 12 |
| $\Phi_{age}$ | $p_{age,dist}$ | $\Psi_{age}$ | 4322.65 | 13.65 | 0.00 | 10 |
| $\Phi_{age}$ | $p_{age,dist}$ | $\Psi_{age,year}$ | 4326.78 | 19.52 | 0.00 | 15 |
| $\Phi_{age}$ | $p_{age,dist}$ | $\Psi_{.}$ | 4448.63 | 139.63 | 0.00 | 9 |

$\Phi,$ survival; $p$*,* recapture; Ψ, transition; QAIC_C_, quasi-likelihood Akaike’s information criterion corrected for small sample size; *K*, number of parameters; *w_i_*, Akaike’s model weights; *age*, age-class structure including first-year age-class and pooled age-class after first-year; *dist*, state-dependent variation in natal dispersal distance; *year*, annual variation; (.), indicates a constant for parameter.
