# Supplementary material for: Juvenile Survival in a Neotropical Migratory Songbird Is Lower than Expected
Source: PLoS One. 2013 Feb 8;8(2):e56059. doi: 10.1371/journal.pone.0056059 (PMC3568049; doi:10.1371/journal.pone.0056059)
Supplement: Table S2 — Model selection to estimate recapture probabilities for Prothonotary Warblers, Protonotaria citrea, in southern Illinois, USA, 2004–10. (DOCX) [file pone.0056059.s003.docx]

Table S2. Model selection to estimate recapture probabilities for Prothonotary Warblers, *Protonotaria citrea*, in southern Illinois, USA, 2004-10.

| $\Phi$ | Ψ | $p$ | QAIC_C_ | ΔQAIC_C_ | *w_i_* | *K* |
| --- | --- | --- | --- | --- | --- | --- |
| $\Phi_{age}$ | $\Psi_{dist}$ | $p_{age,year}$ | 4297.39 | 0 | 0.98 | 13 |
| $\Phi_{age}$ | $\Psi_{dist}$ | $p_{age}$ | 4305.50 | 8.11 | 0.02 | 8 |
| $\Phi_{age}$ | $\Psi_{dist}$ | $p_{age,dist}$ | 4309.01 | 11.61 | 0.00 | 11 |
| $\Phi_{age}$ | $\Psi_{dist}$ | $p_{age,year,dist}$ | 4315.69 | 18.30 | 0.00 | 31 |
| $\Phi_{age}$ | $\Psi_{dist}$ | $p_{year}$ | 4336.64 | 39.24 | 0.00 | 12 |
| $\Phi_{age}$ | $\Psi_{dist}$ | $p_{.}$ | 4339.80 | 42.41 | 0.00 | 7 |

$\Phi,$ survival; Ψ, transition; $p$*,* recapture; QAIC_C_, quasi-likelihood Akaike’s information criterion corrected for small sample size; *K*, number of parameters; *w_i_*, Akaike’s model weights; *age*, age-class structure including first-year age-class and pooled age-class after first-year; *dist*, state-dependent variation in natal dispersal distance; *year*, annual variation; (.), indicates a constant for parameter.
